# Quantitative assessment of brain sparing effect in fetal growth restriction: structural evidence from fetal MRI

**DOI:** 10.3389/fnins.2026.1889399

**Published:** 2026-09-07

**Authors:** Jida Wang, Weizeng Zheng, Yuehong Cheng, Meixiang Deng, Kui Li, Xiaomin Liu, Zhuying Chen

**Affiliations:** Department of Radiology, Women’s Hospital School of Medicine Zhejiang University, Zhejiang Provincial Clinical Research Center for Obstetrics and Gynecology, Hangzhou, Zhejiang, China

**Keywords:** fetal brain sparing, fetal growth restriction, fetal MRI, small for gestational age, volume ratio

## Abstract

**Objective:**

To differentiate brain-sparing patterns in fetal growth restriction (FGR) versus small-for-gestational-age (SGA) fetuses using MRI volumetry.

**Methods:**

This retrospective study analyzed whole-body MRI data from 378 fetuses (28–40 weeks’ gestation) collected between 2018 and 2025, comprising 308 normal controls, 31 SGA, and 39 FGR cases. Fetal body and brain volumes were segmented using a nnU-Net model. Adjusted linear regression models analyzed growth trajectories of body volume, brain volume, and the brain-to-body volume ratio. Diagnostic performance was evaluated using the area under the curve (AUC).

**Results:**

SGA fetuses exhibited an 18.2% lower body growth rate than controls (156.1 vs. 190.9 mL/week) but comparable brain growth, indicating preserved brain-sparing. Conversely, FGR fetuses showed significant reductions in both brain (−18.4%) and body (−15.8%) growth rates. Consequently, the brain-to-body volume ratio decreased rapidly in FGR (−0.005/week vs. −0.002/week in controls), signaling a failure of the brain-sparing mechanism. The ratio model achieved AUCs of 0.989 (Normal vs. FGR), 0.94 (Normal vs. SGA), and 0.883 (SGA vs. FGR).

**Conclusion:**

SGA is characterized by restricted body growth with relative brain preservation, confirming active brain-sparing. FGR involves significant global restriction and a disproportionately decreasing volume ratio, signifying a breakdown of this mechanism. The brain-to-body volume ratio is a sensitive biomarker for distinguishing these distinct adaptive patterns.

## 1 Introduction

Fetal Growth Restriction (FGR) is a significant obstetric complication where a fetus fails to achieve its genetically predetermined growth potential (Lees et al., 2022), poses significant risks for perinatal morbidity and long-term neurodevelopmental impairment (Lees et al., 2013; Meher et al., 2015; Sacchi et al., 2020). It is most commonly caused by placental insufficiency, which leads to a state of chronic undernutrition and hypoxia (Bamfo and Odibo, 2011; Society for Maternal-Fetal Medicine et al., 2020). In response to the chronic hypoxia and undernutrition characteristic of FGR, the fetus initiates a hemodynamic adaptation known as the “brain sparing” effect, where blood flow is preferentially shunted to vital organs, including the brain (Flood et al., 2014; Giussani, 2016). While this mechanism is intended to be protective, its presence indicates that the fetus is under significant physiological stress, and the protective effect is imperfect and gradually evolves into a detrimental response (Allison et al., 2026). Contrary to full protection, many studies report that the brain-sparing effects associated with reduced total brain volume and altered cortical volume and structure, decreased total number of cells and myelination deficits (Miller et al., 2016; Arthurs et al., 2017; Meijerink et al., 2025).

The diagnosis of fetal growth restriction (FGR) usually uses Delphi consensus, which is determined by statistical deviation of fetal size from population-based reference standards, defining those biometry below the 10th percentile as small for gestational age (SGA), while SGA with abnormal ultrasound Doppler and those below the 3rd percentile are defined as FGR (Gordijn et al., 2016). However, the majority of SGA pregnancies are associated with a good prognosis compared to FGR pregnancies (Dashe et al., 2000; Unterscheider et al., 2013). The diagnosis of brain-sparing effect in fetal growth restriction heavily relies on Doppler ultrasound, particularly measurements of the middle cerebral artery pulse index (PI) and cerebroplacental ratio (Beukers et al., 2017; Romero and Hernandez-Andrade, 2015). Similarly, there is a significant lack of international consensus on precise diagnostic thresholds for brain-sparing, with various cutoff values being used in research and clinical practice (Conde-Agudelo et al., 2018; Lees et al., 2020), and no relevant studies have involved quantifying the brain-sparing effect in SGA and FGR.

Fetal magnetic resonance imaging (MRI) has become indispensable, offering unparalleled anatomical detail and soft tissue contrast for precise diagnosis (Kadji et al., 2019). Unlike ultrasound, MRI is not limited by maternal obesity, fetal position, late gestation, or oligohydramnios (Aertsen et al., 2019). Furthermore, advances in MRI technology have significantly improved scan speed. Fast 3D imaging can now be acquired in a single breath-hold, reducing the impact of fetal motion, maternal respiratory artifacts, and slice-to-slice displacement inherent in conventional 2D sequences, thereby enabling accurate volumetric measurements of the entire fetus and individual organs (Damodaram et al., 2012; Uus et al., 2024).

Recently, deep learning algorithms like nnU-Net (Isensee et al., 2020) have revolutionized medical image segmentation. The open-source nnU-Net is a semantic segmentation framework that automatically adapts its pipeline to a dataset. It analyzes the training data, creates a dataset fingerprint, configures suitable U-Net variants, and provides an end-to-end workflow from preprocessing to training, model selection, and inference. Trained on expert-annotated datasets, the segment models perform precise identification in seconds, establishing a technical foundation for automated fetal MRI volumetry.

## 2 Materials and methods

The protocol for this study was approved by the ethics committee of our hospital (approval number: IRB-20250312-R). Due to the retrospective design of this study, the requirement for written informed consent was waived.

### 2.1 Study population

**Figure 1:**
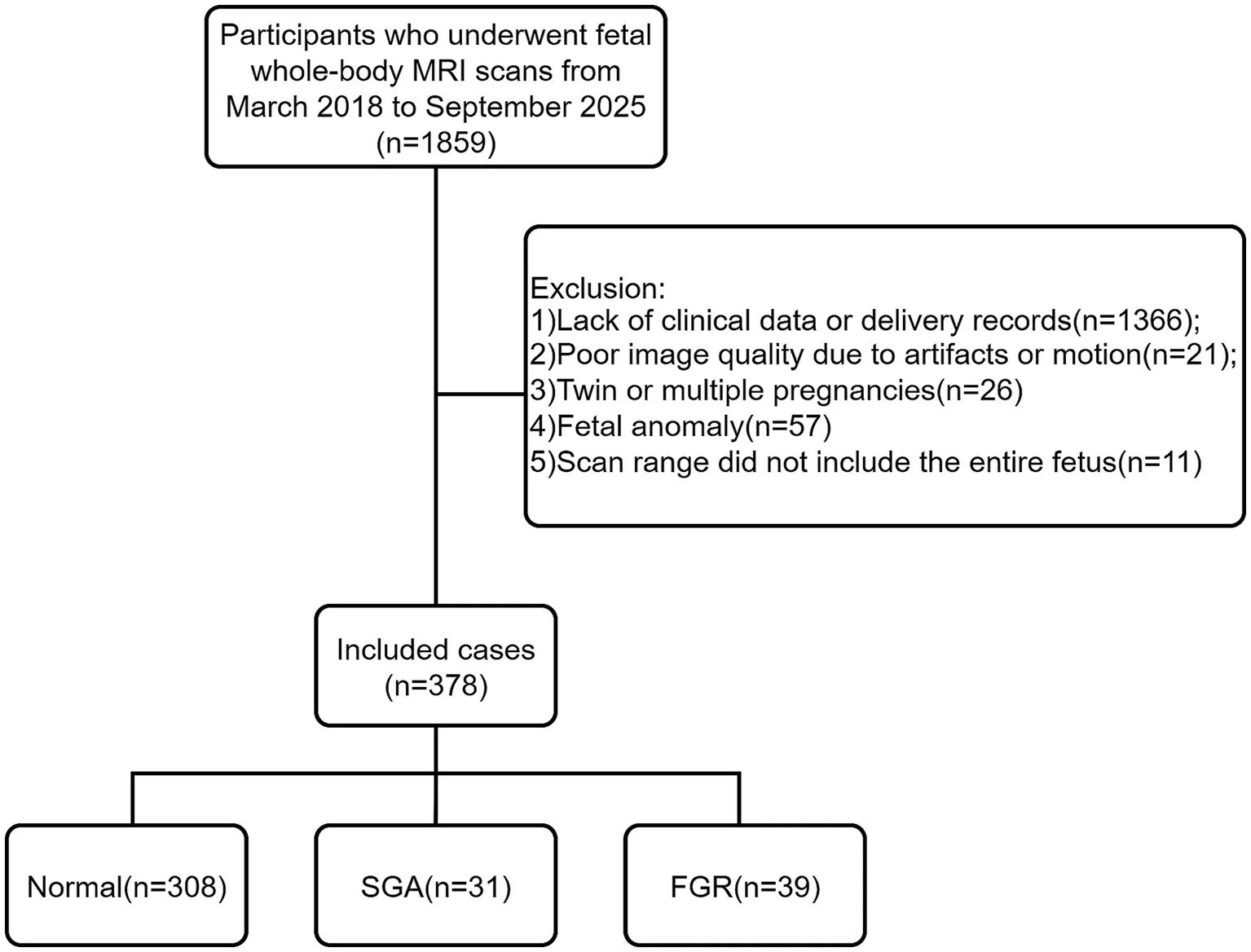
Flowchart of study population selection.

The study cohort included pregnant women at 28–40 weeks of GA who underwent fetal MRI and delivery in our hospital from March 2018 to September 2025. MR images were retrieved from a picture archiving and communication system, and clinical data from an electronic medical record system. The inclusion criteria for cases: (1) singleton pregnancy; (2) birth weight (BW) > 10th percentile of GA, no severe maternal complications during pregnancy, classified into Normal group; (3) BW < 10th percentile of GA, classified into SGA group; (4) SGA with umbilical artery (UA) PI > 95th percentile and BW < 3rd percentile of GA, classified into FGR group; (5) no congenital anomalies; (6) available MRI data. Exclusion criteria: (1) missing delivery records; (2) insufficient clinical and prenatal examination data due to incomplete medical records; (3) poor quality MRI images due to artifacts or motion; (4) twin or multiple pregnancies; (5) fetal anomalies; (6) MR scan range did not include the entire fetus. Figure 1 is the detailed data flowchart.

### 2.2 MRI protocol

MR images were acquired on 1.5-T MR SIGNA HDxt scanner (GE Healthcare, WI, USA) with 8-channel phased-array cardiac coil, and 3-T MR SIGNA Premier scanner (GE Healthcare, WI, USA) with 30-channel Air coil and 60-channel spine posterior Air coil, participants were placed in the supine or left lateral decubitus position depending on comfort level. A 3D fast imaging employing steady-state acquisition (3D-FIESTA) sequence with a breath-hold was used to image fetal whole body, and the specific parameters were as follows: (1) 1.5-T, repetition time, 2.6–3.3 ms; echo time, 1.3 ms; flip angle, 60°; matrix size, 256 × 224; field-of-view, 36–40 cm^2^; slice thickness, 3–3.5 mm; number of slices, 96; and acquisition time, 15–18 s; (2) 3-T, repetition time, 2.6–3.8 ms; echo time, 1.8 ms; flip angle, 60°; matrix size, 224 × 224; field-of-view, 40 cm^2^; slice thickness, 3–4 mm; number of slices, 60; and acquisition time, 16–19 s.

### 2.3 Image segmentation and volume calculation

All fetal MR images were retrieved from the Picture Archiving and Communication System and preprocessed with N4 bias field correction and resampling to a 1:1:1 resolution. Two radiologists (with 13 and 15 years of MRI diagnostic experience, respectively) randomly selected 30 images and manually segmented the fetal body and brain using 3D-Slicer software (version 5.8.1; https://github.com/Slicer/Slicer). Any discrepancies were resolved through mutual discussion to reach consensus. The consensus manual segmentation result was considered the ground truth. Furthermore, to assess intra-observer agreement, one of the radiologists re-segmented the images 1 month later.

Manually segmented images and masks were fed into the nn-UNet V2 pipeline (version 2.2; https://github.com/MIC-DKFZ/nnUNet/) to train fetal whole-body and brain segmentation models, respectively. The training configuration used the “3d_fullres” mode and the default nnU-Net V2 sampling strategy. Each model was trained for 250 epochs with 5-fold cross-validation. The training process was executed on a single NVIDIA RTX 4070Ti super GPU equipped with 16 GB of graphics memory, using PyTorch (version 2.7.1, https://pytorch.org/get-started/locally/) and CUDA (version 12.8; https://developer.nvidia.com/cuda-12-8-0-download-archive). The volume represented by the segmentation mask was calculated using SimpleITK (version 2.1.1.1; https://github.com/SimpleITK/SimpleITK).

### 2.4 Statistical analysis

Statistical analysis was conducted by R software (version 4.5.1; http://www.R-project.org). In descriptive statistics, continuous variables were presented as mean ± standard deviation, and categorical variables were presented as frequency and percentage. In the between-group comparison, the ANOVA test was used for the continuous variables with normal distribution, and the Kruskal–Wallis test was used for the non-normal distribution of data. The chi-square test and the Fisher exact test were used for the categorical variables.

Intraclass correlation coefficients (ICCs) were used to assess inter- and intra-observer agreement in manually segmented volumes. The performance of nnU-net was evaluated by the Dice similarity coefficient (DSC, ranging from 0 to 1).

Three multiple linear regression models with interaction (body volume, brain volume, brain-to-body volume ratio) were established to adjust for the effects of the following factors on fetal volume: GA (in weeks) at MR, maternal age, body mass index (BMI), gravidity and parity as continuous variables; fetal sex as categorical variables; and group and GA as interaction terms. Adjusted volume growth slopes and predicted values at key GA were calculated. Intergroup comparisons were performed using non-parametric tests. Receiver operating characteristics (ROC) curves determine sensitivity and specificity to evaluate the diagnostic efficiency of the model. In addition, intergroup heterogeneity analysis for each covariate was also conducted. A *p*-value less than 0.05 was considered to be statistically significant.

## 3 Results

### 3.1 Study population and baseline characteristics

A total of 378 participants were selected (308 Normal, 31 SGA, 39 FGR), the baseline characteristics of the study population in each group is shown in Table 1. Brain volume, body volume, and volume ratios showed statistically significant differences between groups, with the FGR group having an earlier delivery GA compared to the other groups.

**Table 1:** Baseline characteristics of the study population in each group.

Variable	Normal *N* = 308^1^	Abnormal		*p*-value^2^
		SGA *N* = 31^1^	FGR *N* = 39^1^	
Brain_volume (mL)	277.67 ± 69.49	236.75 ± 61.62	211.40 ± 47.61	<0.001*
Body_volume (mL)	2,338.22 ± 657.34	1,779.77 ± 492.45	1,437.24 ± 443.15	<0.001*
Brain_body_ratio	0.121 ± 0.013	0.134 ± 0.011	0.152 ± 0.026	<0.001*
GA_MR (week)	34.55 ± 3.25	33.61 ± 3.11	33.05 ± 2.62	0.008*
Fetal_sex				0.6
Female	170 (55.2%)	20 (64.5%)	22 (56.4%)	
Male	138 (44.8%)	11 (35.5%)	17 (43.6%)	
Maternal_age (year)	31.30 ± 3.97	31.42 ± 5.40	30.79 ± 3.84	0.9
Maternal_BMI	26.14 ± 3.04	25.12 ± 3.55	25.29 ± 2.98	0.084
GA_delivery (week)	38.52 ± 1.35	38.16 ± 1.24	35.56 ± 2.96	<0.001*
Gravidity	1.96 ± 1.30	2.13 ± 1.41	1.85 ± 1.27	0.6
Parity	1.32 ± 0.57	1.32 ± 0.48	1.23 ± 0.43	0.7

^1^Mean ± SD; *n* (%); ^2^Kruskal–Wallis test or ANOVA test; Pearson’s Chi-squared test; *p < 0.05 for differences among Normal, SGA and FGR groups; GA, gestational age; MR, magnetic resonance; BMI, body mass index.

### 3.2 Segmentation model performance

**Figure 2:**
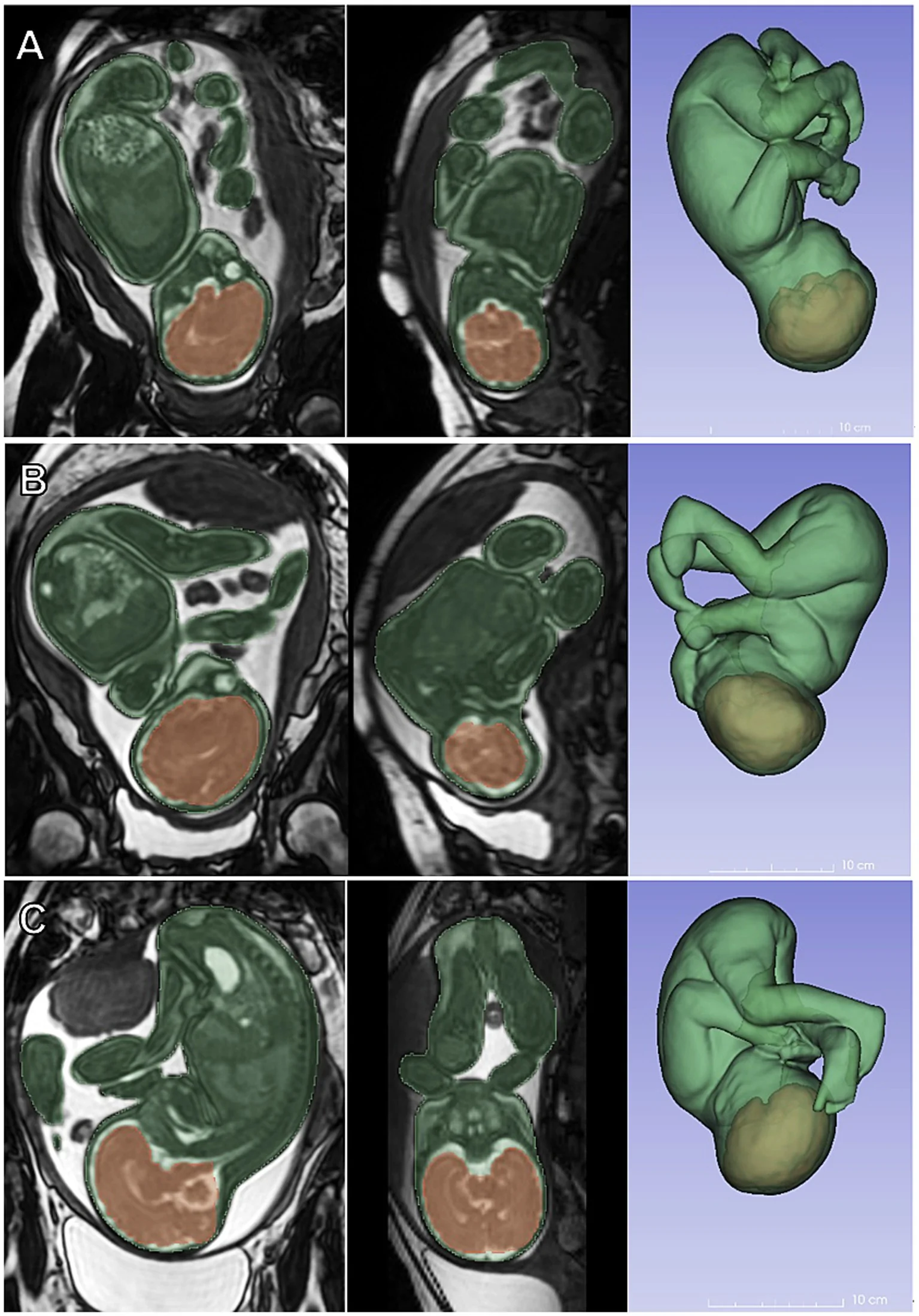
Fetal MR images, masks, and 3D segmentation visualization. **(A)** Normal case: volume ratio = 0.116; **(B)** SGA case: volume ratio = 0.128; **(C)** FGR case: volume ratio = 0.142.

Intra- and inter-observer agreement for manual segmentation of the fetal body and brain was excellent, with inter-observer ICCs of 0.943 and 0.969, and intra-observer ICCs of 0.981 and 0.958, respectively. The automatic segmentation inference time of nnU-Net was 60–95 s per case, which is substantially faster than manual segmentation (30–60 min per case). The average DSC for the fetal body and brain segmentation models were 0.955 and 0.957, respectively. The trained weights of fetal body and brain segmentation is available at https://github.com/hlwjd/FetalSeg. Figure 2 shows the coronal and sagittal images, masks, and 3D segmentation visualization of example cases in the Normal, SGA, and FGR groups.

### 3.3 Fetal growth trajectories

**Figure 3:**
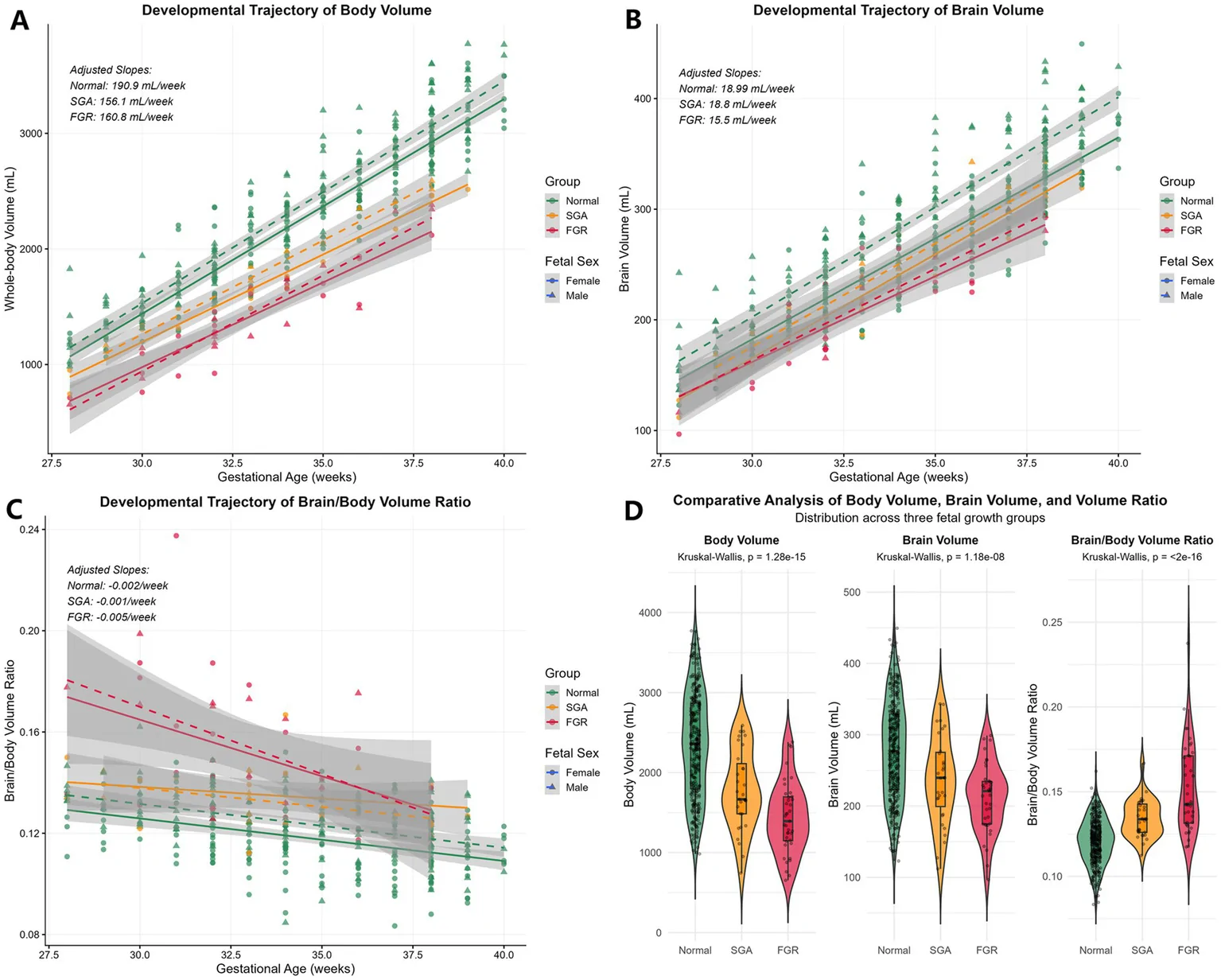
Scatter plots and violin box plots of body volume, brain volume, and volume ratio. **(A–C)** Circles and solid lines represent the scatter plots and regression line for female fetuses, and triangles and dotted lines represent the scatter plots and regression line for male fetuses. Shading indicates the 95% confidence interval (CI). **(D)** The Kruskal–Wallis test showed statistically significant differences in body volume, brain volume, and volume ratio.

**Figure 4:**
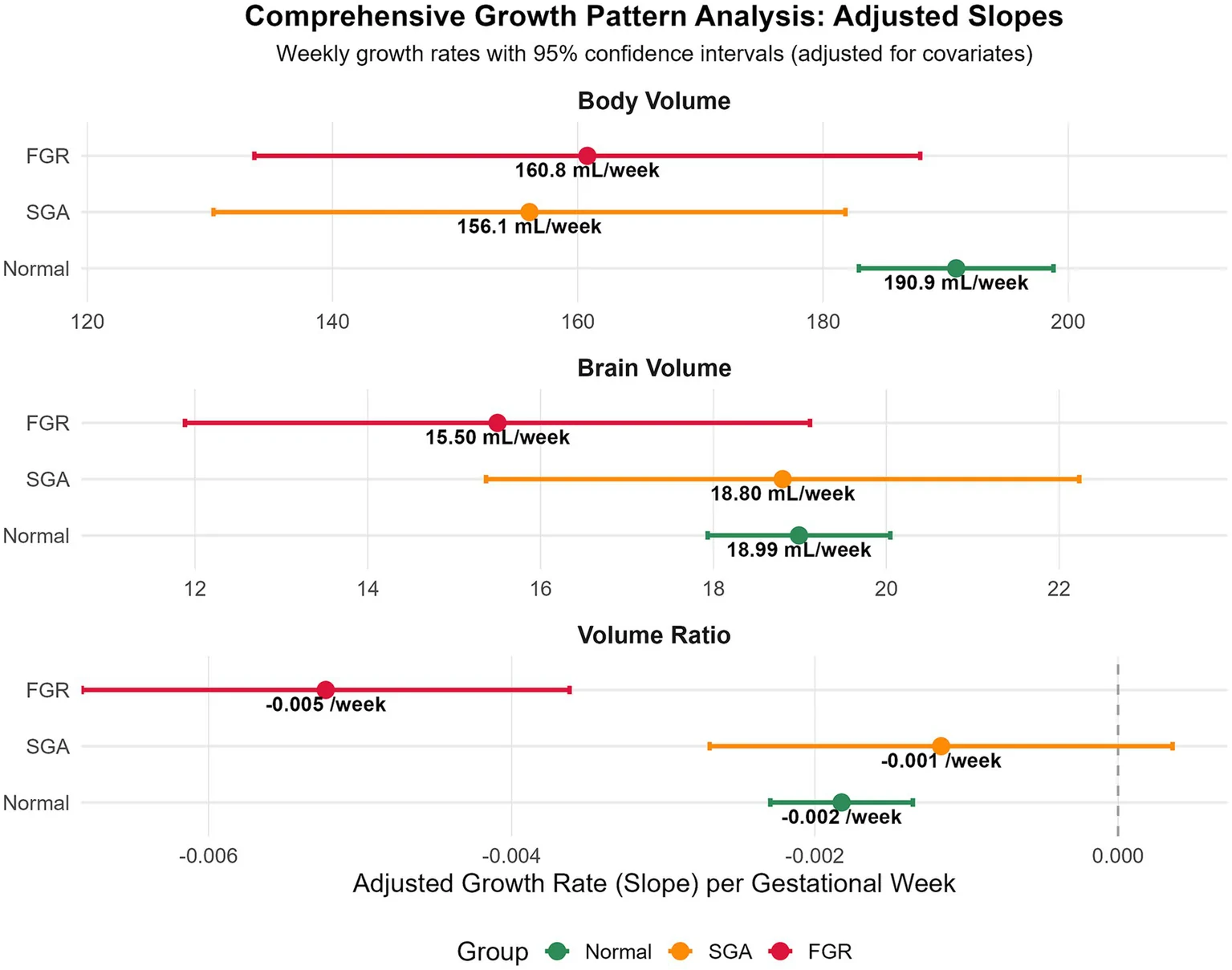
Growth slope forest plot of body volume, brain volume, and brain/body volume ratio.

Three linear regression models were established to characterize the growth trajectories of total body volume, brain volume, and the brain-to-body volume ratio across gestational weeks for the Normal, SGA, and FGR groups (Figure 3). The body volume model demonstrated an excellent fit (R^2^ = 0.901, adjusted R^2^ = 0.899). The adjusted growth slopes were 190.9 mL/week (95% CI: 182.9 to 198.8) for the Normal group, 156.1 mL/week (95% CI: 130.3 to 181.8) for the SGA group, and 160.8 mL/week (95% CI: 133.6 to 187.9) for the FGR group. The model predicted distinct volume trajectories, the Normal group consistently exhibited the highest volumes, followed by the SGA and FGR groups. The brain volume model also showed a strong fit (R^2^ = 0.83, adjusted R^2^ = 0.825). The growth slope for the Normal group was 18.99 mL/week (95% CI: 17.93 to 20.05). This slope was similar for the SGA group at 18.8 mL/week (95% CI: 15.37 to 22.23) but was notably lower for the FGR group at 15.5 mL/week (95% CI: 11.89 to 19.12). The model for the brain-to-body volume ratio had a moderate fit (R^2^ = 0.482, adjusted R^2^ = 0.468). The growth patterns of the ratio differed among the groups. The Normal group showed a steady decrease of −0.002 per week (95% CI: −0.002 to −0.001). The SGA group displayed a non-significant trend (slope: −0.001 per week, 95% CI: −0.003 to 0), whereas the FGR group exhibited a significantly steeper decrease of −0.005 per week (95% CI: −0.007 to −0.004). Figure 4 and Table 2 show the adjusted growth slope and intergroup differences.

**Table 2:** Adjusted growth slopes with 95% CI by group and intergroup differences (*p < 0.05).

Growth slope	Normal	SGA	FGR	SGA vs Normal	FGR vs Normal
Body volume (mL/week)	190.9 (182.9–198.8)	156.1 (130.3–181.8)	160.8 (133.6–187.9)	↓18.2%*	↓15.8%
Brain volume (mL/week)	18.99 (17.93–20.05)	18.80 (15.37–22.23)	15.50 (11.89–19.12)	↓1.0%	↓18.4%
Brain/body ratio (/week)	−0.002 (−0.002 to −0.001)	−0.001 (−0.003 to 0.000)	−0.005 (−0.007 to −0.004)	↓36.0%	↑186.6%*

The volume prediction values at key GA are presented in Table 3 and Supplementary Figure S1. The SGA group showed varying degrees of brain sparing effect from 30 to 38 weeks; in contrast, the FGR group showed delayed brain growth but relatively preserved body growth.

**Table 3:** Predicted volume values and preservation percentiles at key gestational weeks.

Gestational weeks	Volume measure	Normal	SGA	FGR	SGA Preservation	FGR Preservation
30 weeks	Body volume (mL)	1,416	1,185	908	83.7%	64.2%
	Brain volume (mL)	180	159	153	88.2%	84.9%
	Brain/body ratio	0.127	0.136	0.166	106.9%	130.2%
34 weeks	Body volume (mL)	2,179	1809	1,552	83.0%	71.2%
	Brain volume (mL)	256	234	215	91.4%	83.9%
	Brain/body ratio	0.120	0.131	0.145	109.6%	120.7%
38 weeks	Body volume (mL)	2,943	2,433	2,195	82.7%	74.6%
	Brain volume (mL)	332	310	277	93.2%	83.4%
	Brain/body ratio	0.112	0.127	0.124	112.5%	110.0%

Preservation = predicted value in SGA or FGR ÷ predicted value in Normal × 100%. For Brain/Body Ratio, values >100% indicate a higher brain-to-body ratio reflecting brain-sparing in growth-restricted fetuses.

### 3.4 Diagnostic performance of the brain-to-body volume ratio

**Figure 5:**
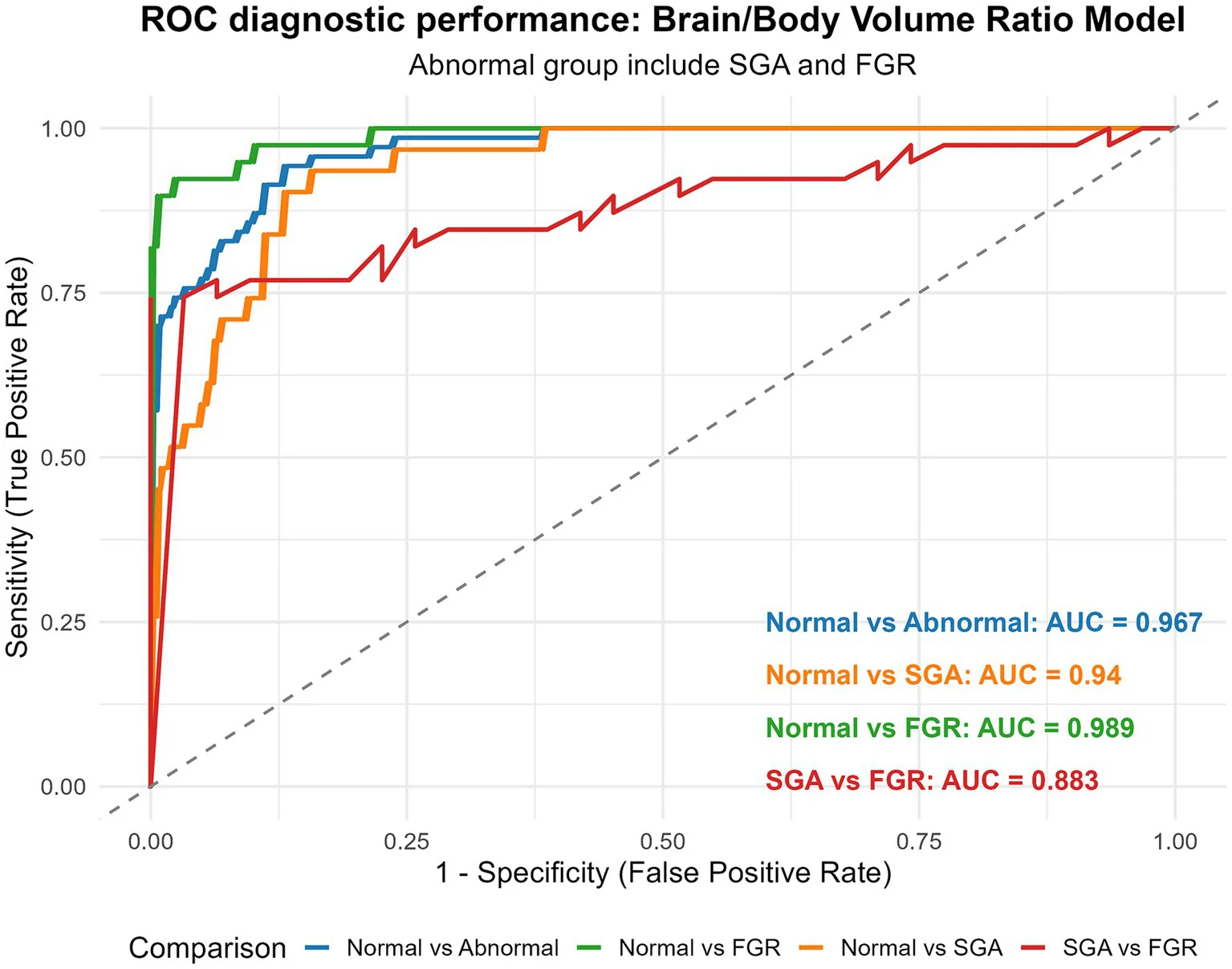
ROC curve assessing the performance of fetal brain/body volume ratios.

Brain-to-body volume ratio demonstrated exceptional diagnostic performance for identifying FGR, achieving an AUC of 0.989 (95% CI: 0.976–1) with a sensitivity of 92.3% and specificity of 97.7% at the optimal threshold of 0.134. This performance significantly surpassed that of body volume (AUC 0.875, sensitivity 87.2%, specificity 73.4%) and brain volume (AUC 0.796, sensitivity 84.6%, specificity 65.3%) measurements (both *p* < 0.001). The diagnostic advantage of the brain-to-body ratio was consistently observed across all classification tasks. For Normal vs. Abnormal differentiation, it achieved an AUC of 0.967 with 94.3% sensitivity and 87% specificity. In distinguishing Normal vs. SGA, it attained an AUC of 0.940 with 93.5% sensitivity and 84.4% specificity. Even for the challenging SGA vs. FGR classification, the ratio model maintained robust performance (AUC 0.883, sensitivity 74.4%, specificity 100%). In contrast, both body volume and brain volume models showed comparatively lower sensitivity and specificity across all classification scenarios. (Table 4; Figure 5; Supplementary Figures S2, S3).

**Table 4:** ROC curve evaluates the diagnostic performance of body volume, brain volume, and brain/body volume ratio.

Comparison	Parameter	AUC (95% CI)	Best Threshold	Sensitivity	Specificity	Youden Index
Normal vs abnormal	Body volume	0.820 (0.773–0.868)	2042.12 mL	0.843	0.656	0.499
	Brain volume	0.745 (0.687–0.802)	249.32 mL	0.771	0.653	0.424
	Brain/body ratio	0.967 (0.949–0.985)	0.129	0.943	0.870	0.813
Normal vs SGA	Body volume	0.751 (0.678–0.823)	2110.34 mL	0.774	0.633	0.407
	Brain volume	0.681 (0.592–0.770)	260.20 mL	0.742	0.591	0.333
	Brain/body ratio	0.940 (0.907–0.973)	0.128	0.935	0.844	0.780
Normal vs FGR	Body volume	0.875 (0.829–0.922)	1873.52 mL	0.872	0.734	0.606
	Brain volume	0.796 (0.735–0.857)	248.83 mL	0.846	0.653	0.499
	Brain/body ratio	0.989 (0.976–1.000)	0.134	0.923	0.977	0.900
SGA vs FGR	Body volume	0.701 (0.573–0.830)	1630.77 mL	0.718	0.742	0.460
	Brain volume	0.641 (0.504–0.778)	230.69 mL	0.718	0.613	0.331
	Brain/body ratio	0.883 (0.802–0.965)	0.144	0.744	1.000	0.744

### 3.5 Heterogeneity and covariate analyses

Significant fetal sex differences in brain volume (*p* = 0.025) and brain-to-body ratio (*p* < 0.001) were observed only in the normal group, while maternal BMI also showed statistically significant differences in body volume (*p* < 0.001) and brain-to-body ratio (*p* < 0.001) only in the normal group, suggesting that fetal growth restriction may override typical heterogeneity patterns (Supplementary Tables S1, S2). Maternal age, gravidity and parity influenced approximately 2% of volume variance, with no observed statistically significant differences between groups.

## 4 Discussion

Our study provides quantitative in-utero evidence supporting the distinct pathophysiological pathways underlying SGA and FGR. The SGA group exhibited a growth pattern characterized by relative brain sparing, with brain growth largely preserved (99.0% of the normal rate) while body growth was substantially restricted (81.8% of the normal rate). In stark contrast, the FGR group showed a failure of this compensatory brain-sparing mechanism. Both brain (81.6% of the normal rate) and body (84.2% of the normal rate) growth were significantly compromised, with the brain being more severely affected than the body, while the growth slope of the brain-to-body volume ratio is lower than that of the normal group. Crucially, the brain-to-body volume ratio emerged as a superior discriminator, demonstrating exceptional diagnostic performance in distinguishing both FGR (AUC: 0.989) and SGA (AUC: 0.940) from normal fetuses. Furthermore, we observed that sexual dimorphism was present in the normal development group, but this sex-related difference was absent in both the SGA and FGR groups, suggesting that pathological conditions may override or mask physiological sex-specific developmental divergence.

The three groups differed significantly in GA at both MRI examination (*p* = 0.008) and delivery (*p* < 0.001), with FGR fetuses delivered approximately 3 weeks earlier than the other groups. To account for this, GA at MRI was explicitly modeled as a covariate together with its interaction with group in all linear regression models, such that all between-group comparisons represent adjusted estimates at a common GA. Nevertheless, the earlier delivery in FGR reflects the clinical decision to expedite delivery in the setting of suspected placental insufficiency, which should be considered when interpreting the group differences in growth trajectories.

Our findings align with and extend the existing literature on fetal adaptive programming. The observed preservation of brain volume in SGA fetuses is consistent with the well-established “brain-sparing effect,” an adaptive response to mild-to-moderate nutrient and oxygen deprivation that prioritizes blood flow to vital organs such as the brain, heart, and adrenal glands while sacrificing other body parts (Baschat, 2011; Figueras and Gratacós, 2014). This study quantitatively confirms that this protective mechanism remains operational in SGA fetuses.

More importantly, our data provide, to our knowledge, the first volumetric MRI-based evidence that this compensatory mechanism is fundamentally disrupted in FGR. The significant reduction in both brain and body volumes in the FGR group suggests a progression beyond the fetus’s adaptive capacity. This supports the concept that FGR represents a more severe, decompensated state of placental insufficiency compared to SGA (Lees et al., 2020; Steller et al., 2022), and the brain-sparing is not merely a protective phenomenon but transitions from an adaptive state to a decompensated state as the duration and severity of hypoxemia increase (Allison et al., 2026). Previous studies have typically focused only on the overall or partial volume of the fetal brain (Meijerink et al., 2025), or on ultrasound Doppler indices of cerebral blood vessels to measure the varying degrees of brain sparing effects (Ciobanu et al., 2019; Romero and Hernandez-Andrade, 2015; Turan et al., 2008), without characterizing the extent of damage to this mechanism. Consequently, the brain-to-body volume ratio proves to be a powerful biomarker, outperforming isolated body or brain volume measurements by directly quantifying the breakdown of the brain-sparing physiology.

The divergent growth patterns between SGA and FGR can be explained by the severity of placental dysfunction (Kovo et al., 2013; Sun et al., 2020). In SGA, a milder insult likely triggers successful adaptive hemodynamic changes, including blood flow redistribution, which safeguards brain development at the expense of somatic growth (Giussani, 2016). In contrast, FGR likely results from severe, early-onset placental insufficiency that overwhelms these compensatory mechanisms, leading to global hypoxemia that the fetus cannot circumvent, thereby affecting the brain (Maunu et al., 2007)—the most oxygen-sensitive organ—in addition to the body.

Another noteworthy finding is the presence of sex differences in body volume and brain volume in the normal group, which aligns with the known sexual dimorphism in typical fetal development (Raznahan et al., 2011; Villar et al., 2014). However, this difference disappears in the SGA group and FGR group, forming a striking contrast. The disappearance of this sex difference may suggest that severe placental pathology triggers a general, gender-independent maladaptive developmental program, overriding the typical hormonal and genetic influences that drive dimorphic growth.

The disappearance of this sex-related difference in growth-restricted fetuses may be explained by the higher metabolic demand of male fetuses, which are typically larger and grow faster than females. Under conditions of placental insufficiency, this greater nutritional and oxygen requirement may render male fetuses more vulnerable to growth restriction, thereby masking the physiological sexual dimorphism. However, whether this effect is mediated by placental insufficiency or reflects sex-specific developmental programs remains to be determined in future studies with placental pathology and serial volumetric measurements.

The findings from this study carry significant potential for clinical translation. Firstly, the brain-to-body volume ratio offers a powerful tool for the early and accurate prenatal diagnosis of true FGR, enabling it to be distinguished from SGA fetuses with relatively good prognosis. This is crucial for risk stratification and avoiding the unnecessary intervention in benign SGA cases.

Secondly, the assessment of brain-sparing status via the volume ratio may serve as a prognostic indicator for postnatal neurodevelopmental outcomes. Fetuses with failed brain-sparing (i.e., low volume ratio) might be at a substantially higher risk for adverse neurological sequelae, warranting closer surveillance and early intervention strategies.

Finally, our results underscore the necessity for differential clinical management. Recognizing SGA and FGR as distinct entities implies that SGA fetuses may require primarily surveillance for well-being, while FGR fetuses demand intensive monitoring and well-timed delivery to prevent in-utero demise or long-term impairment.

In addition, the automated segmentation model we developed demonstrates rapid inference and excellent segmentation performance, significantly reducing analysis time and providing better conditions for clinical application and promotion.

## 5 Limitations

Several limitations of our study should be acknowledged. First, the sample size was unbalanced, with the SGA and FGR groups being smaller than the Normal group, which may affect the generalizability of the findings. Second, the cross-sectional design precludes the assessment of longitudinal growth trajectories and their correlation with postnatal outcomes. Future studies with serial scans and long-term neurodevelopmental follow-up are essential. Third, the study lacked direct assessment of placental function and concurrent Doppler ultrasonography parameters, which would have provided a more comprehensive pathophysiological context for the volumetric findings.

## 6 Conclusion

This study provides quantitative structural evidence for different patterns of brain sparing effects in FGR and SGA through fetal MRI brain-to-body volume ratio measurement, offering an important tool for precise classification and prognostic evaluation of fetal growth restriction.

## Data Availability

The raw data supporting the conclusions of this article will be made available by the authors, without undue reservation.
