# Supplementary material for: Quantitative assessment of brain sparing effect in fetal growth restriction: structural evidence from fetal MRI
Source: Front Neurosci. 2026 Sep 7;20:1889399. doi: 10.3389/fnins.2026.1889399 (PMC13592097; doi:10.3389/fnins.2026.1889399)
Supplement: Supplementary file 1 [file Table_1.docx]

Table S1. Sex effects on body volume, brain volume, and brain/body volume ratio within each fetal growth group. Mean difference with Wilcoxon rank-sum P value (nonparametric, original data).

| **Group** | **Body Volume (mL)** | **Brain Volume (mL)** | **Brain/Body Ratio** |
| --- | --- | --- | --- |
| Normal (n=308) | +40.5 (P=0.694) | +19.4 (P=0.025) | +0.0062 (P=<0.001) |
| SGA (n=31) | +90.1 (P=0.730) | +7.9 (P=0.951) | -0.0024 (P=0.823) |
| FGR (n=39) | +91.1 (P=0.812) | +11.7 (P=0.528) | +0.0004 (P=0.900) |
