# Supplementary material for: Quantitative assessment of brain sparing effect in fetal growth restriction: structural evidence from fetal MRI
Source: Front Neurosci. 2026 Sep 7;20:1889399. doi: 10.3389/fnins.2026.1889399 (PMC13592097; doi:10.3389/fnins.2026.1889399)
Supplement: Supplementary file 2 [file Table_2.docx]

Table S2. Maternal BMI effects (per 5-unit increase) on body volume, brain volume, and brain/body volume ratio within each fetal growth group. Linear regression estimates adjusted for gestational age and fetal sex.

| **Group** | **Body Volume (mL)** | **Brain Volume (mL)** | **Brain/Body Ratio** |
| --- | --- | --- | --- |
| Normal (n=308) | +112.9 (95%CI +70.3 to +155.6, P=<0.001) | -2.9 (95%CI -8.6 to +2.8, P=0.315) | -0.0065 (95%CI -0.0085 to -0.0044, P=<0.001) |
| SGA (n=31) | -28.2 (95%CI -79.1 to +22.6, P=0.286) | +4.8 (95%CI -5.8 to +15.3, P=0.382) | +0.0046 (95%CI -0.0007 to +0.0100, P=0.099) |
| FGR (n=39) | -72.9 (95%CI -169.9 to +24.2, P=0.150) | -5.6 (95%CI -18.6 to +7.3, P=0.401) | +0.0045 (95%CI -0.0090 to +0.0179, P=0.522) |
